# Involvement of SIK3 in Glucose and Lipid Homeostasis in Mice

**DOI:** 10.1371/journal.pone.0037803

**Published:** 2012-05-25

**Authors:** Tatsuya Uebi, Yumi Itoh, Osamu Hatano, Ayako Kumagai, Masato Sanosaka, Tsutomu Sasaki, Satoru Sasagawa, Junko Doi, Keita Tatsumi, Kuniko Mitamura, Eiichi Morii, Katsuyuki Aozasa, Tomohiro Kawamura, Meinoshin Okumura, Jun Nakae, Hajime Takikawa, Toshio Fukusato, Minako Koura, Mayumi Nish, Anders Hamsten, Angela Silveira, Alejandro M. Bertorello, Kazuo Kitagawa, Yasuo Nagaoka, Hidehisa Kawahara, Takeshi Tomonaga, Tetsuji Naka, Shigeo Ikegawa, Noriyuki Tsumaki, Junichiro Matsuda, Hiroshi Takemori

**Affiliations:** 1 Laboratory of Cell Signaling and Metabolic Disease, National Institute of Biomedical Innovation, Ibaraki, Osaka, Japan; 2 Department of Anatomy, Nara Medical University, Nara, Japan; 3 Department of Life Science and Biotechnology, Kansai University, Suita, Osaka, Japan; 4 Department of Neurology, Osaka University Graduate School of Medicine, Osaka, Japan; 5 Department of Bone and Cartilage Biology, Osaka University Graduate School of Medicine, Osaka, Japan; 6 Food and Nutrition, Senri Kinran University, Osaka, Japan; 7 Department of Laboratory Medicine, Osaka University Graduate School of Medicine, Osaka, Japan; 8 Faculty of Pharmaceutical Sciences, Kinki University, Osaka, Japan; 9 Department of Pathology, Osaka University Graduate School of Medicine, Osaka, Japan; 10 Department of General Thoracic Surgery, Osaka University Graduate School of Medicine, Osaka, Japan; 11 Frontier Medicine on Metabolic Syndrome, Keio University School of Medicine, Tokyo, Japan; 12 Department of Medicine, Teikyo University School of Medicine, Tokyo, Japan; 13 Department of Pathology, Teikyo University School of Medicine, Tokyo, Japan; 14 Animal Models for Human Diseases, National Institute of Biomedical Innovation, Ibaraki, Osaka, Japan; 15 Cardiovascular Genetics and Genomics, Atherosclerosis Research Unit, Karolinska Institutet, CMM, Karolinska University Hospital-Solna, Stockholm, Sweden; 16 Membrane Signaling Networks, Atherosclerosis Research Unit, Karolinska Institutet, CMM, Karolinska University Hospital-Solna, Stockholm, Sweden; 17 Laboratory of Proteome Research, National Institute of Biomedical Innovation, Ibaraki, Osaka, Japan; 18 Laboratory for Immune Signal, National Institute of Biomedical Innovation, Ibaraki, Osaka, Japan; 19 Department of Cell Growth and Differentiation, Center for iPS Cell Research and Application, Kyoto University, Kyoto, Japan; Clermont Université, France

## Abstract

Salt-inducible kinase 3 (SIK3), an AMP-activated protein kinase-related kinase, is induced in the murine liver after the consumption of a diet rich in fat, sucrose, and cholesterol. To examine whether SIK3 can modulate glucose and lipid metabolism in the liver, we analyzed phenotypes of SIK3-deficent mice. *Sik3* ^−/−^ mice have a malnourished the phenotype (*i.e.*, lipodystrophy, hypolipidemia, hypoglycemia, and hyper-insulin sensitivity) accompanied by cholestasis and cholelithiasis. The hypoglycemic and hyper-insulin-sensitive phenotypes may be due to reduced energy storage, which is represented by the low expression levels of mRNA for components of the fatty acid synthesis pathways in the liver. The biliary disorders in *Sik3* ^−/−^ mice are associated with the dysregulation of gene expression programs that respond to nutritional stresses and are probably regulated by nuclear receptors. Retinoic acid plays a role in cholesterol and bile acid homeostasis, wheras ALDH1a which produces retinoic acid, is expressed at low levels in *Sik3* ^−/−^ mice. Lipid metabolism disorders in *Sik3* ^−/−^ mice are ameliorated by the treatment with 9-cis-retinoic acid. In conclusion, SIK3 is a novel energy regulator that modulates cholesterol and bile acid metabolism by coupling with retinoid metabolism, and may alter the size of energy storage in mice.

## Introduction

Cholesterol has diverse functions in eukaryotes, *e.g.*, as a cell membrane component and a source of hormones and bile acid (BA). Dysregulation of cholesterol metabolism is involved in a variety of disease, such as dyslipidemia, cardiovascular disease, and obesity [1]. The liver X receptor (LXR) is a nuclear receptor that binds to target DNA elements by forming a heterodimer complex with the retinoid X receptor (RXR) [2], [3]. Excess cholesterol is sensed by LXRs as their ligands, and active LXR-RXR complexes promote the gene expression of cholesterol-catabolic enzymes (*e.g.,* cytochrome P450 family 7A [CYP7A], which catabolizes cholesterol to BA in the liver) and cholesterol transporters, *e.g.,* ATP-cassette G5 (ABCG5) and G8 in the liver and ABCA1 in the peripheral tissues. LXR also up-regulates hepatic fatty acid (FA) synthesis by inducing the expression of sterol regulatory element-binding protein 1c (SREBP1c) [4].

BA is also multifunctional molecules with a role in the digestive tract. The impairment of bile flow by bile duct lesions and cholelithiasis causes the retention of excess amounts of BA (cholestasis) and leads to chronic hepatitis. The farnesoid X receptor (FXR) senses BA as its ligand, forms a complex with RXR, and up-regulates gene expression to lower the level of BA in the liver [5] by inducing the bile salt export pump (BSEP) and small heterodimer partner (SHP), which suppresses *Cyp7a* expression [6]. Excess levels of the BA pool also enhance energy expenditure and suppression of FA synthesis [7]. Meanwhile, a reduction of the BA pool by the activation of FXR induces obesity and hyperglycemia [8], suggesting that cholesterol-BA homeostasis is important for lipid and glucose metabolism.

9-*Cis* retinoic acid (9*-*cis*-*RA), an endogenous RXR ligand, is synthesized from vitamin A [9]. Vitamin A deficiency or RXR inhibition results in reduced LXR and FXR activity, which can lead to hepatic cholestasis [1]. Conversely, dysregulation of the metabolism of vitamin A to 9-cis*-*RA induces resistance to diet-induced obesity and type 2 diabetes in mice [10]. Because vitamin A absorption by enterocytes requires BA, BA homeostasis is tightly coupled with vitamin A metabolism [11].

Salt-inducible kinase (SIK), a member of the 5′-AMP-activated protein kinase (AMPK)-related kinase family, has 3 isoforms and regulates gene expression in various cells [12]. For example, SIK1 inhibits steroidogenic gene expression in the adrenal glands and gluconeogenic gene expression programs in the liver by repressing the cAMP response element (CRE)-binding protein (CREB) transcription factor [13], [14] [15]–[16]. Meanwhile, SIK2 suppresses insulin-dependent thermogenic gene expression in brown adipose tissue [17]. In addition, in mice with a disrupted *Sik2* gene, downregulation of SIK2 expression confers resistance to oxidative stresses after brain ischemia [18] and enhances melanogenesis in melanocytes after ultraviolet irradiation [19], [20]. These SIK2-dependent physiological events are also explained by the modulation of CREB activity.

When CREB is phosphorylated at Ser133 in its kinase-inducible domain by upstream activating kinases, such as protein kinase A (PKA) and Ca^2+^/calmodulin-dependent kinase I/IV (CaMKI/IV), it recruits coactivators, *e.g.*, CREB-binding protein and p300 and induces CRE-dependent transcription [21]. The other CREB-specific coactivator, *i.e.*, CREB regulated transcription coactivator (CRTC or TORC), also activates CREB in response to PKA and CaMKI/IV [22], [23]. In contrast to CREB, CRTC is inactivated by phosphorylation and is sequestered in the cytoplasm of unstimulated cells [24]. SIK1 and SIK2 are among the CRTC kinases that are involved in SIK-mediated inhibition of CREB [25]. Recently, p300 was also reported to be a mediator of SIK signaling in hepatocytes [26]. SIK2 inhibits the coactivation activity of p300 by phosphorylating Ser89, which prevents carbohydrate response element-binding protein-dependent hepatic steatosis in mice.

In addition to CREB and p300 repression, SIK1 induces hypertrophic action in the muscles by inhibiting class 2a histone deacetylase (HDAC) and then upregulating MEF2C transcription activity [27]. Recently, SIK2 was also found to inactivate class 2a HDAC in *Drosophila*, which results in the accumulation of FA in the fat body of insects and confers resistance to starvation [28]. These observations suggest that like AMPK, SIK1 and SIK2 may play important roles in the regulation of metabolic or stress responses.

SIK3 is also capable of regulating CREB activity in cultured cells under overexpression [29] or *in vitro* conditions [30]. Recently, we found that mice with a disrupted *Sik3* gene showed dwarfism because of the impairment of chondrocyte hypertrophy during skeletal development, which was accompanied by disinactivation of class 2a HDAC in the cartilage [31]. However, SIK3 phenotypes in adult mice, especially those related to energy metabolism, have not yet been elucidated.

Here, we report the induction of *Sik3* mRNA in the mouse liver after the consumption of a high-fat diet supplemented with excess cholesterol. Phenotyping of adult *Sik3* ^−/−^ mice suggested that SIK3 is a novel regulator of glucose-lipid metabolism in the liver that maintains cholesterol-BA homeostasis along with the regulation of lipid storage size.

## Results

### Sik3^−/−^ Mice Exhibit a Lipodystrophic Phenotype

Factors affecting body size and longevity in model organisms, such as *Caenorhabditis elegans* and *Drosophila*, often play important roles in the regulation of energy metabolism in mammals. The same may hold true for SIK. The *C. elegans kin-29* (ortholog of SIK) mutant shows increased longevity and small body size [32], while *Drosophila* expressing reduced levels of SIK2 acquired resistance to oxidative stress and starvation [33]. We also found that *Sik2* ^−/−^ mice show resistance to brain ischemia [18]; however, *Sik2* ^−/−^ mice are apparently normal in terms of body weight regulation [19].

**Figure 1:**
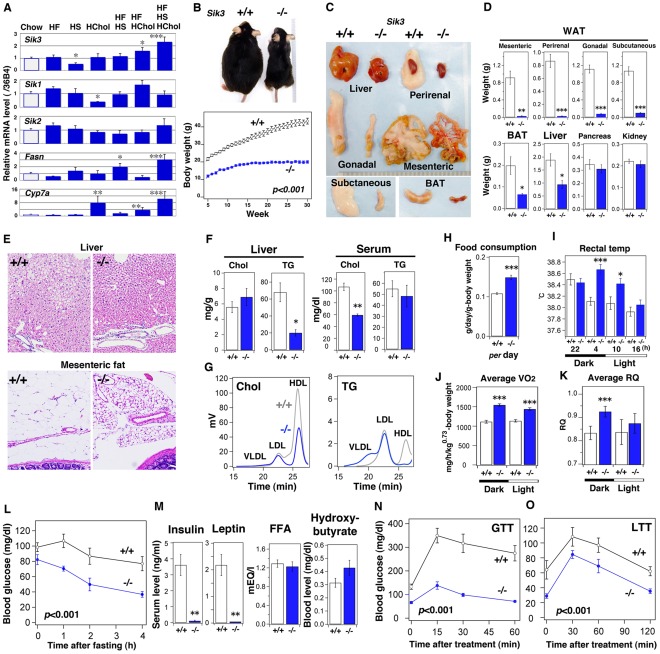
*Sik3* ^−/−^ mice are lean, hypolipidemic, and hypoglycemic. (A) C57BL/6 mice (male: n = 4) were fed various diets (HF, high fat; HS, high sucrose; HChol, high cholesterol) for 2 weeks, and liver mRNA was examined by quantitative PCR. *, **, and *** indicate *p*<0.05, <0.01, and <0.001, respectively. Means and SEM are shown. (B) The body weight of male mice (n = 6) was monitored. All data points show *p*<0.001. (C) One-year-old male mice (n = 5) were sacrificed (scale: 1 mm), and the indicated tissues were weighed (D). (E) Histology of liver and mesenteric fatty tissue is shown. Each magnification is the same. (F) Cholesterol (Chol) and triglycerides (TG) in the liver and serum were measured (n = 5). (G) Serum cholesterol and TG were separated using FPLC. (H) The food consumption of each group (n = 12). (I) Rectal temperature (n = 12). (J) Oxygen consumption (VO_2_, voluntarily O_2_ consumption) and (K) average respiratory quotient (RQ) during the day and night (n = 5). (L) Mice (n = 5) were fasted and their blood glucose levels were monitored at the indicated time points. All data points show *p*<0.001. (M) After 4-h fasting, the serum levels of insulin, leptin, free fatty acid (FFA), and ß-hydroxybutyrate were measured. (N) After 4-h fasting, glucose (1.5 g/kg) was intraperitoneally injected (GTT, glucose tolerance test) and blood glucose levels were monitored (n = 5). (O) After 24-h fasting, lactate (1.5 g/kg) was injected intraperitoneally (LTT, lactate tolerance test; n = 5).

To reevaluate individual SIK isoforms in the regulation of nutrient metabolism, normal C57BL/6J mice were fed with a variety of diets, and we examined their mRNA levels were examined. Interestingly, *Sik3* mRNA was strongly induced in the livers of the mice fed a high-fat/high-sucrose/high-cholesterol (HF/HS/HChol) diet. This up-regulation was accompanied by the induction of mRNA for metabolic enzymes such as *FA synthase* (*Fasn*) and *Cyp7a* (Figure 1A). These results led us to investigate of the metabolic profiles of *Sik3* knockout mice [31].

Although *Sik3* ^−/−^ mice were indistinguishable from wild-type mice just after birth, most of the knockout (KO) mice died on the first day (Figure S1A). Caesarean delivery failed to prevent the early death of *Sik3* ^−/−^ mice. Because *Sik3* ^−/−^ mice had skeletal abnormalities, their early death was probably due to respiratory failure caused by thoracic dystrophy [31]. However, the transgenic expression of SIK3 in the cartilage of *Sik3* ^−/−^ mice failed to prevent their early death despite its correction of the skeletal abnormalities (no *Sik3* ^−/−^ mouse survived out of seventeen weanling mice derived from the matings between *Sik3* ^−/−^ females and *Sik3* ^−/−^ :: *Col11a2-hSik3* males).

The *Sik3* ^−/−^ mice that survived the first day could be weaned (Figure S1B), but their body weight was obviously less than that of the wild-type mice (male; Figure 1B). This was also the case with the females. We dissected 1-year-old mice and found that the lean phenotype of *Sik3* ^−/−^ mice was attributed to the liver and adipose tissues, especially mesenteric and perirenal fat (Figure 1C and D). Small but substantial amounts of gonadal and subcutaneous fat and brown adipose tissue were observed in *Sik3* ^−/−^ mice. Hematoxylin and eosin (HE) staining suggested that the small fat pads were probably due to the small size of the adipocytes (Figure 1E and S1C). The low levels of liver TG in *Sik3* ^−/−^ mice might have prevented the development of fatty liver (Figure 1E and F), while total cholesterol levels were low in the serum of *Sik3* ^−/−^ mice. Fast protein liquid chromatography (FPLC) analysis of serum lipids indicated that *Sik3* ^−/−^ mice exhibited hypo-high density lipoprotein (HDL) cholesterolemia (Figure 1G).

To elucidate the causes of the lipodystrophic phenotype of *Sik3* ^−/−^ mice, we compared the energy balance between wild-type and *Sik3* ^−/−^ mice. *Sik3* ^−/−^ mice consumed more food than the wild-type mice (Figure 1H), while the rate of digestion and absorption in the intestine appeared normal (Figure S1D). The rectal temperature of *Sik3* ^−/−^ mice was higher than that of the wild-type mice (Figure 1I), which might correlate with the high levels of the O_2_ consumption (VO_2_: voluntary O_2_ consumption) observed in *Sik3* ^−/−^ mice (Figure 1J). The high respiratory quotient (RQ) value of the *Sik3* ^−/−^ mice was well explained by the insufficient fat storage followed by reduced fat utilization observed in these mice (Figure 1K). These results suggested that the lipodystrophic phenotype of *Sik3* ^−/−^ mice might be a result of their high rates of energy consumption.

We also examined secondary parameters such as blood glucose levels. In the fed condition, the *Sik3* ^−/−^ mice had slightly lower blood glucose levels than the wild-type mice, and the levels quickly decreased after fasting (Figure 1L). After a 4-h fast, the *Sik3* ^−/−^ mice had significantly lower serum insulin and leptin levels than the wild-type mice (Figure 1M), while no obvious differences were observed in free FA or ketone body (ß-hydroxybutyrate) levels. Given the enhanced food-consumption of *Sik3* ^−/−^ mice, the apparently enhanced insulin- and leptin-action may be restricted to the peripheral tissues. Although we suspected thyrotoxicosis, the levels of circulating thyroid hormones did not differ between the 2 genotypes (Figure S1E).

**Figure 2:**
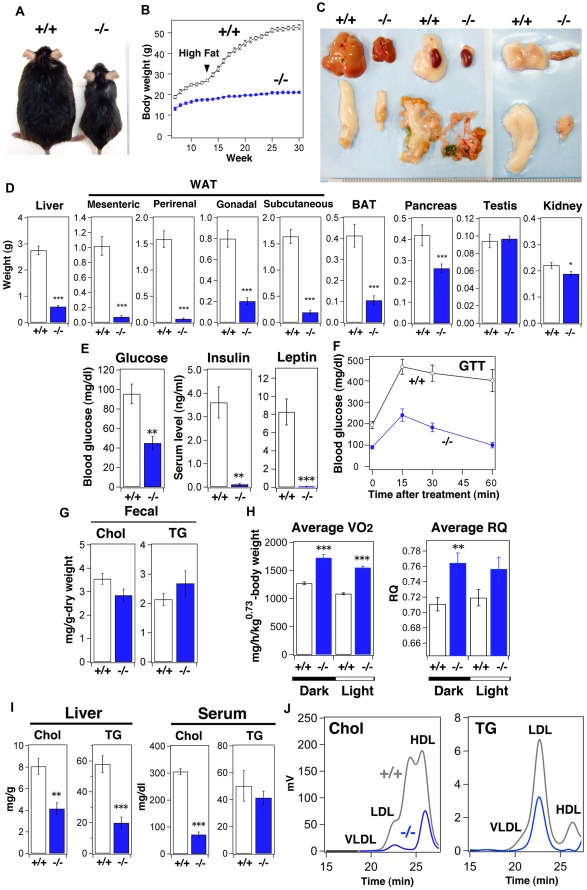
*Sik3* ^−/−^ mice are resistant to a high-fat diet. (A) A representative image of male mice after high-fat (60% of calories) feeding. Mice (n = 6) were fed with a high-fat diet for 12–30 weeks and their body weights were monitored every week (B). Means and SEM are shown. All data points indicate *p*<0.001. (C) Representative photos of tissues (scale, 1 mm). (D) Tissue weights are shown. *, **, and *** indicate *p*<0.05, <0.01, and <0.001, respectively. (E) Levels of blood glucose, insulin, and leptin measured after 4-h fasting. (F) Glucose tolerance test (1.5 g/kg) performed after 4-h fasting. (G) Cholesterol and triglyceride content in feces (n = 3 cages). (H) Oxygen consumption (VO_2_, voluntarily O_2_ consumption) of each group (n = 6) was monitored. Respiration quotient (RQ) during the day and night. (I) Cholesterol (Chol) and triglycerides (TG) in the liver and serum were measured (n = 6). (J) Serum Chol and TG were separated using FPLC.

*Sik3* ^−/−^ mice exhibited enhanced glucose tolerance (GTT) (Figure 1N). When *Sik3* ^−/−^ mice were treated with insulin (ITT), their blood glucose levels decreased like those of the wild-type mice (Figure S1F). Once the *Sik3* ^−/−^ mice were supplied exogenously with an energy source, such as lactate (lactate tolerance test), they were able to produce glucose efficiently (Figure 1O), suggesting that the hypoglycemia of *Sik3* ^−/−^ mice may be due to a lack of energy storage followed by an enhanced insulin response. This was also the case under the high-fat diet feeding condition (Figure 2A–J). Of particular note is that accumulation of cholesterol was suppressed in the livers of *Sik3* ^−/−^ mice (Figure 2I), and the ratio of LDL-cholesterol to HDL-cholesterol was decreased in the serum of *Sik3* ^−/−^ mice with a reduction of TG content in the very low-density lipoprotein (VLDL) fraction (Figure 2J).

Which tissue is responsible for the phenotypes of the *Sik3* ^−/−^ mice? To address this question, we measured the body weight of heterozygous (*Sik3* ^+/−^ ) mice. The body weight curve of the *Sik3* ^+/−^ mice overlapped with that of the wild-type mice (Figure S2A). Curiously, the mRNA and protein levels of SIK3 in the livers of *Sik3* ^+/−^ mice were as high as those in wild-type mice (Figure S2B), though the specific levels of *Sik3* mRNA did not differ between parenchymal and non-parenchymal cells (Figure S2C). Given that parenchymal cells are the major population of the liver, we surmised that the liver, probably parenchymal cells, may be one of the responsible tissues/cells for the phenotypes of the *Sik3* ^−/−^ mice.

Mice with disrupted genes for *Mark2* [34] or *Mark3* [35], which are other members of the AMPK family, have been found to be resistant to diet-induced obesity due to enhanced glucose-utilization in the brown adipose tissue. However, the mRNA expression levels of genes related to energy expenditure, such as *Ppargc1a* and *Ucp1*, in brown adipose tissue were not different between wild-type and *Sik3* ^−/−^ mice.

Moreover, an *in vitro* adipocyte differentiation experiment using gonadal fat indicated that the preadipocytes of *Sik3* ^−/−^ mice possessed a higher capability to differentiate into adipocytes than those from wild-type mice (Figure S2D), which might correlate with the high serum level of adiponectin in *Sik3* ^−/−^ mice (Figure S2E). Given the expression level of *Sik3* mRNA (Figure S2B), we surmised that the lipodystrophic phenotype of *Sik3* ^−/−^ mice might be caused by the impairments of the liver rather than the adipose tissues.

### Signaling States in the Livers of Sik3^−/−^ Mice

**Figure 3:**
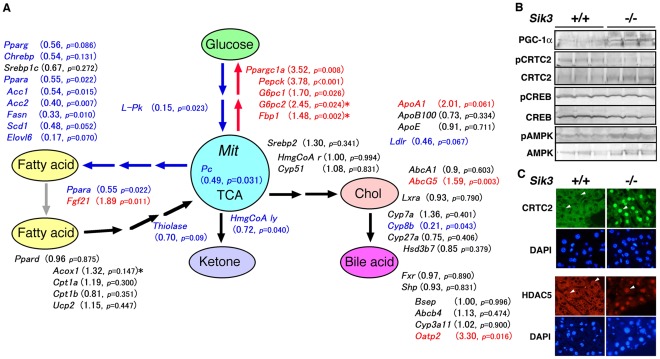
Gene expression profile in the liver. (A) One-year-old male mice (n = 5) were fasted for 4 h, and the liver mRNA levels were measured using quantitative polymerase chain reaction (qPCR). Red and blue indicate the up- and down-regulated genes in *Sik3* ^−/−^ mice, respectively. +, fold increase; -, fold decrease. The threshold is set at *p = *0.1. The values marked with an asterisk (*) were obtained using PCR-array kits (n = 3). The abbreviations for the genes and the PCR primers used are listed in Table S2. Mit, mitochondria; TCA, tricarboxylic acid cycle. (B) Intracellular signaling molecules and their activation status in the liver were examined by western blot analysis. (C) Immunohistochemical analysis of SIK3 substrates (CRTC2 and HDAC5) in the liver.

The gene expression profile of the liver (Figure 3A) indicated that the pathway from glycolysis to FA synthesis was down-regulated in *Sik3* ^−/−^ mice, while the glyconeogenic pathway was up-regulated. *Sik3* ^−/−^ mice expressed high levels of *Fgf21* mRNA, suggesting an adaptive response to starvation; however, its promoting pathway, *i.e.*, the peroxisome proliferator-activated receptor alpha (PPARα) pathway, was down-regulated. Lack of FA storage and the uncoupling of FGF21 from the PPARα pathway in *Sik3* ^−/−^ mice may result in the failure to induce ß-oxidation followed by ketogenesis [36].

We also examined the state of signaling molecules. The high level of PGC-1α protein in the liver of *Sik3* ^−/−^ mice was accompanied by the dephosphorylation of CRTC2 (Figure 3B) despite there being no significant difference in the status of CREB. Interestingly, the level of another CRTC2 kinase, AMPK [15], and of its activated phosphorylated form (pThr172) were also high in the livers of *Sik3* ^−/−^ mice. Immunohistochemical analyses revealed the enhanced accumulation of CRTC2 in the nuclei of *Sik3* ^−/−^ mice hepatocytes (Figure 3C). In addition, HDAC5, another SIK/AMPK substrate [37], [38], also accumulated in the nuclei of liver cells in *Sik3* ^−/−^ mice, suggesting that AMPK is unable to compensate for the deficiency of SIK3 in hepatocytes.

### Sik3^−/−^ Mice are Unable to Adapt to Cholesterol

Adiponectin had been found to promote fat accumulation in adipose tissues and to improve insulin sensitivity in leptin-resistant mice [39], which could explain the hypoglycemic phenotype of *Sik3* ^−/−^ mice, but not their lipodystrophy. What are the unknown factors? Interestingly, little, if any, changes were found in the mRNA levels of the cholesterol and BA metabolic genes in *Sik3* ^−/−^ mice (Figure 3A) and irregular expression patterns were observed, *i.e.*, *ApoA1* and *Abcg5* were up-regulated, while *Cyp8b* was strongly suppressed. Moreover, hepatic *Sik3* mRNA expression is induced by a high-fat diet supplemented with high-cholesterol (Figure 1A). Therefore, we decided to examine effects of cholesterol (with fat) on *Sik3* ^−/−^ mice by challenging the mice with a high-cholesterol diet (HF/HS/HChol).

**Figure 4:**
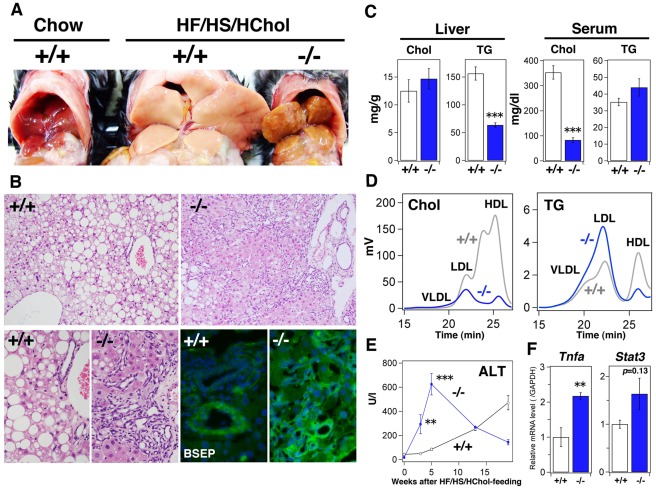
*Sik3* ^−/−^ mice are less tolerant to a cholesterol-containing high-fat diet. (A) Male mice were fed a high-fat and high-sucrose diet supplemented with 2% cholesterol (HF/HS/HChol) for 4 months (12–30 weeks) and then sacrificed (n = 6). (B) HE staining of the liver (sets at the *upper* and *lower left*), BSEP-staining (*lower right*: BSEP is green and nuclei are blue (DAPI)). The magnification is the same in each set. (C) Cholesterol and TG levels in the liver and serum were measured (n = 6). *** indicates *p*<0.001. Means and SEM are shown. (D) FPLC analysis of serum lipids. (E) Serum levels of alanine aminotransferase (ALT) were monitored at the indicated time points. ** indicates *p*<0.01. (F) Quantitative polymerase chain reaction analysis of inflammatory molecules (tumor necrosis factor-α and STAT3) in the liver.

After 4 months, the wild-type mice developed fatty liver, while the livers of *Sik3* ^−/−^ mice had surface asperity and turned yellow (Figure 4A). HE staining revealed the enhanced formation of a dilated canalicular structure in the livers of *Sik3* ^−/−^ mice (Fig 4B, *upper* and *lower left*), and these structures were highly positive for BSEP-immunoreactive signals (*green signals* in Figure 4B *lower right*). Liver and serum lipid levels were increased after feeding with the HF/HS/HChol diet; however, they were not significantly different from the levels observed when wild-type and *Sik3* ^−/−^ mice were fed a chow diet (compare Figure 1F to Figure 4C). The ratio of LDL-cholesterol to HDL-cholesterol in *Sik3* ^−/−^ mice was reversed after feeding with the cholesterol-containing diet (compare Figure 1G to Figure 4D), and was accompanied by an increase in TG content in the LDL fraction in *Sik3* ^−/−^ mice.

The fluctuations in serum alanine amino transferase (ALT) levels suggested that the livers of *Sik3* ^−/−^ mice were damaged soon after feeding and lost their normal function, *e.g.*, ALT production, after 5 weeks (Figure 4E). While liver injury in the wild-type mice progressed gradually, probably due to fatty liver, the degree of latent liver injury after 4-month of receiving the high-cholesterol diet was higher in *Sik3^−/−^* mice, possibly because of the higher mRNA expression levels of inflammatory factors in the livers of *Sik3* ^−/−^ mice (Figure 4F).

**Figure 5:**
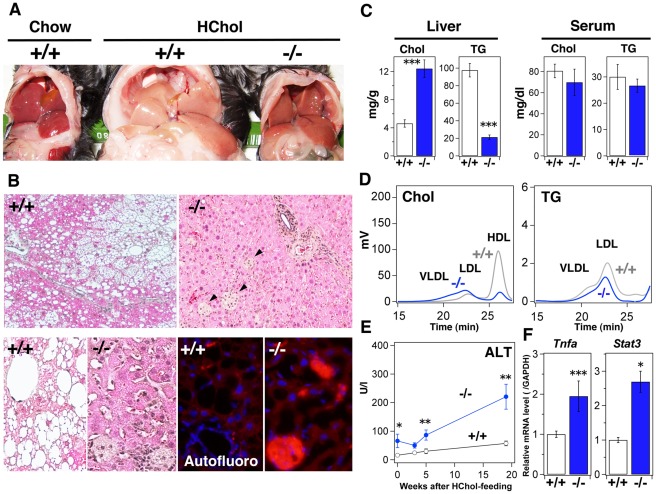
Cholesterol accumulation in the livers of *Sik3* ^−/−^ mice after feeding with a high-cholesterol diet. (A) Male mice were fed a 2% cholesterol diet for 4 months (12–30 weeks) and then sacrificed (n = 5). (B) HE staining of the liver (sets at the *upper* and *lower left*). The arrows indicate eosin-negative foci which with autofluorescence (*lower right*: red, and nuclei are blue (DAPI)). The magnification is the same in each set. (C) Cholesterol and TG levels in the liver and serum were measured (n = 5). *** indicates *p*<0.001. Means and SEM are shown. (D) FPLC analysis of serum lipids. (E) Serum levels of alanine aminotransferase (ALT) were monitored at the indicated time points. * and ** indicate *p*<0.05 and *p*<0.01, respectively. (F) Quantitative polymerase chain reaction analysis of inflammatory molecules in the liver.

To focus on effects of cholesterol alone, *Sik3* ^−/−^ mice were challenged with a high-cholesterol (2%) diet according to the same schedule as the HF/HS/HChol diet. The liver abnormalities of *Sik3* ^−/−^ mice were barely visible on the surface (Figure 5A); however, a number of foci that were negative for eosin-staining (Figure 5B, arrows) were detected in the livers of *Sik3* ^−/−^ mice. These foci might be enriched in cholesterol derivatives, because we observed strong autofluorescence in the frozen sections (Figure 5B *lower right*), and the level of hepatic cholesterol was higher in *Sik3* ^−/−^ mice than in wild-type mice (Figure 5C). A small numbers of dilated canalicular structures were again observed in the *Sik3^−/−^* mice liver (Figure 5B *lower left*).

The patterns of serum lipids in *Sik3* ^−/−^ mice fed with the high-cholesterol diet were almost the same as those of *Sik3* ^−/−^ mice fed with the HF/HS/HChol diet. Liver injury in *Sik3* ^−/−^ mice progressed gradually (Figure 5E), and we observed high mRNA expression levels of inflammatory factors in the liver of *Sik3* ^−/−^ mice (Figure 5F).

**Figure 6:**
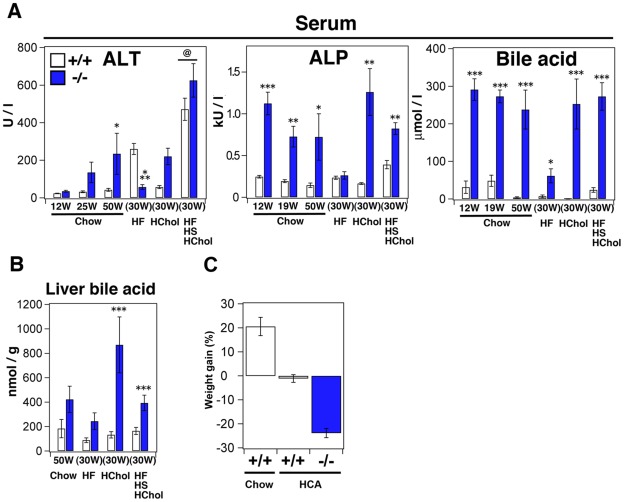
Excess bile acids in the serum and liver of *Sik3* ^−/−^ mice. (A) The serum levels of ALT, alkaline phosphatase, and bile acids are shown. The age at serum collection of the mice fed a chow diet (n = 4–6) are shown, while those of mice fed an HF, HChol, or HF/HS/HChol diet (n = 5–6) was 30 weeks (feeding 12–30 weeks). @ in the ALT panel indicates the maximum values for the results shown in Figure 4E. (B) Bile acids were extracted from the liver (n = 3) and normalized by liver weigh. (C) Gain of body weight after feeding mice with a high cholic acid (CA)-containing diet. Wild type (n = 6) and *Sik3* ^−/−^ mice (n = 5) were fed a diet supplemented with 0.25% CA for 1 month. For the control group, wild-type mice (n = 9) were fed with a chow diet.

Why were the livers of *Sik3* ^−/−^ mice injured after the consumption of the high-cholesterol diet? Are there any hints to explain the lipodystrophic phenotype of these mice? To address these questions, we reevaluated blood biochemical markers for the liver and biliary duct systems. Even when *Sik3* ^−/−^ mice were fed a chow diet, their serum ALT levels gradually increased with age (Figure 6A). However, the high-fat diet (as evidenced by the dissection of a 30-week-old mouse) protected the livers of *Sik3* ^−/−^ mice from the injuries caused by aging as well as by fatty liver. Conversely, once cholesterol is added to the diet, this protection may become invalid. As the serum alkaline phosphatase (ALP) and BA levels were continuously high in *Sik3* ^−/−^ mice, except when under the high-fat diet, we hypothesized that the dysregulation of BA metabolism followed by hepatic cholestasis (Figure 6B) might be the cause of the hepatic injuries.

In addition, to test whether high levels of BA could suppress body weight gain, the mice were fed a high-cholic acid (CA) diet for 1 month. As shown in Figure 6C, the high-CA diet completely suppressed the weight gain of wild-type mice and reduced the body weight of *Sik3* ^−/−^ mice, suggesting that dysregulation of BA metabolism might be one of the causes of the lipodystrophic phenotype of *Sik3* ^−/−^ mice.

### Sik3^−/−^ Mice are Unable to Adapt to CA

**Figure 7:**
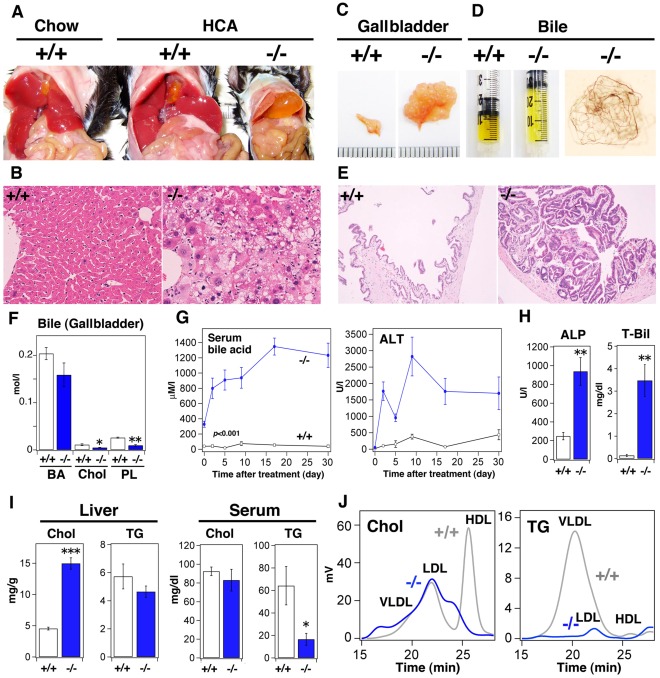
*Sik3* ^−/−^ mice are less tolerant to a cholic acid (CA)-containing diet. (A) Mice (n = 6, but *Sik3* ^−/−^ mouse died before 1 month) were fed a diet supplemented with 0.25% cholic acid for 1 month (12–16 weeks) and then sacrificed. (B) HE staining of the liver (*left*), BSEP staining (*right*: BSEP is green and nuclei are blue (DAPI)). The magnification is the same in each set. (C) Photographs of gallbladders (scale, 1 mm). (D) The color of bile juice and bile sand in the gallbladder. (E) HE staining of the gallbladder. The magnification is the same in both panels. (F) The levels of bile acid (BA), cholesterol (Chol), and phospholipids (PL) in bile juice from the gallbladder were measured. * and ** indicate *p*<0.05 and <0.01, respectively. Means and SEM are shown. (G) Serum BA and alanine aminotransferase (ALT) levels were monitored at the indicated periods. All ALT data points are *p*<0.001, except day 0. (H) Serum alkaline phosphatase (ALP) and total bilirubin (T-Bil) levels were measured. (I) Cholesterol and TG levels in the liver and serum were measured. *** indicates *p*<0.001. (J) FPLC analysis of serum lipids.

To examine the details of the dysregulation of BA metabolism in *Sik3* ^−/−^ mice, we dissected these mice. Their gallbladders of *Sik3* ^−/−^ mice were enlarged, and their livers had become yellow-brown (Figure 7A). HE staining identified hypertrophic hepatocytes with lipid droplets (Figure 7B). The gallbladders of *Sik3* ^−/−^ mice (Figure 7C) were accompanied by hyperplastic mucosal epithelia (Figure 7E).

The volume of bile in the gallbladders of *Sik3* ^−/−^ mice was large, but its color was light (Figure 7D). A good amount of bile sand was also found in the gallbladders of *Sik3* ^−/−^ mice (Figure 7D, *right*). Like FXR-KO mice [40], the deposition of bile sand might be a result of the presence of cholesterol crystals because cholesterol was depleted from the bile of *Sik3* ^−/−^ mice (Figure 7F); this could have been caused by the decreased levels of phospholipids followed by the reduced solubility of bile [41].

High serum BA and ALT levels were observed in *Sik3* ^−/−^ mice on the high-CA diet (Figure 7G). The levels of ALP and total bilirubin were also high in *Sik3* ^−/−^ mice (Figure 7H). The lipid droplets observed in the livers of *Sik3* ^−/−^ mice (Figure 7B) might be composed of cholesterol because cholesterol, and not TG, had accumulated in their livers (Figure 7I). The levels and patterns of serum cholesterol and TG in *Sik3* ^−/−^ mice were also abnormal (Figure 7J); notably, the levels of cholesterol in the VLDL-LDL fraction of the wild-type mice was enhanced by CA feeding, which was more obvious in *Sik3* ^−/−^ mice. Given the severe phenotype caused by CA feeding, we surmised that cholestasis might be the primary phenotype of *Sik3* ^−/−^ mice, and this may then lead to or enhance the other phenotypes, *e.g.*, lipodystrophy and dyslipidemia.

Because most of the *Sik3* ^−/−^ mice were dead on the day of birth (Figure S1A), we examined the livers of embryos. As shown in Figure S3A, hepatocytes in *Sik3* ^−/−^ embryos (E18.5) were not normal; notably, the hepatocytes were of a variable size, and a significant number of multinucleated hepatocytes were observed, suggesting liver damages due to embryonic cholestasis. It was partly true that the BA levels in the livers of *Sik3* ^−/−^ embryos were higher than those of wild-type embryos (Figure S3B). However, when the mice were born, the BA content in the liver of the wild-type mice reached levels equivalent to that of *Sik3* ^−/−^ mice and decreased thereafter, suggesting that hepatic cholestasis might occur in *Sik3* ^−/−^ embryos, but it cannot explain the early death of *Sik3* ^−/−^ mice. Alternatively, we suppose that *Sik3* ^−/−^ neonates may unable to adapt their metabolism to the environmental changes at birth.

### Gene Expression Profile in the Livers of Sik3^−/−^ Mice Fed with Special Diets

We examined mRNA expression in the liver to elucidate the mechanisms involved in the *Sik3* ^−/−^ phenotypes. As shown in Table S1, the resistance to diet-induced obesity in *Sik3* ^−/−^ mice might be explained by the low levels of lipogenic mRNA, *e.g.*, *Fasn* and *Scd1*. Amelioration of hepatic injury in *Sik3* ^−/−^ mice by the high-fat diet was probably due to the down-regulation of cholesterol (*HmgCoAr*) and BA (*Cyp7a*) synthesis.

However, the mRNA expression patterns of mice fed on the high-cholesterol or high-CA diet could not explain the pathogenesis of *Sik3* ^−/−^ *mice*, and some discrepancies remained. When mice were fed with the high-cholesterol diet, no significant difference in the mRNA levels of genes for cholesterol synthesis, *e.g.*, *HmgCoAr*, was observed between wild-type and *Sik3* ^−/−^ mice. The level of *Cyp7a* (bile acid synthesis) mRNA was also the same, despite the high expression level of its repressor (*Shp*). Meanwhile, when the mice were fed with the high-CA diet, *Sik3* ^−/−^ mice expressed lower levels of *Cyp7a* mRNA than the wild-type mice, despite no changes in the levels of *Shp.*

Here, we have to mention some of the problems associated with these gene expression analyses. For example, the mice that were fed the different diets were of different ages. In addition, the special diets were suspected to produce secondary effects, such as hepatic injury. Therefore, we decided to examine gene expression during the acute phase.

### Adaptive Gene Expression is Dysregulated in Sik3^−/−^ Mice

**Figure 8:**
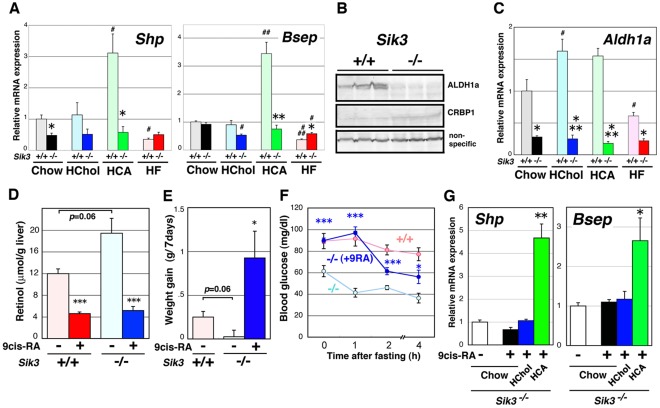
Impairment of cholesterol and bile acid (BA) metabolic gene regulation in *Sik3* ^−/−^ mice. (A) Male mice (12 weeks of age, n = 3) were fed diets supplemented with cholesterol (2%) and cholic acid (CA) (0.25%) for 2 days or with fat (60% of calories) for 2 weeks and then sacrificed. The expression of genes for cholesterol and BA metabolism in the liver was examined using qPCR (normalized by glyceraldehyde 3-phosphate dehydrogenase [*Gapdh*] levels). Significant differences between wild-type and *Sik3* ^−/−^ mice are shown by *, **, and *** for *p*<0.05, <0.01, and <0.001, respectively. # indicates significant differences between the chow and special diet groups. Means and SEM are shown. (B) Western blot analysis of ALDH1a and CRBP1 levels. (C) The levels of *Aldh1a* mRNA in mouse liver (normalized by *Gapdh* levels). (D) Levels of free retinol (vitamin A) in the livers of wild-type and *Sik3* ^−/−^ mice. Mice (12 weeks of age, n = 3) were treated with (+) or without (−) 9-cis*-*RA (8.3 mg/kg, suspended in 1% ethanol) intraperitoneally. After 6 h, the liver was recovered. (E) Mice (wild type, n = 4; *Sik3* ^−/−^ mice, without and with treatment, n = 4 and n = 12, respectively) were treated with 9-cis-RA (4 mg kg^−1^·d^−1^) for 7 days and the weight gain during this period is shown. * indicates a significant difference in the *Sik3* ^−/−^ groups. (F) After 7 days of treatment, the mice in each group were fasted and their blood glucose levels were monitored. Significance was calculated in the *Sik3* ^−/−^ groups. (G) At day 7, the *Sik3* ^−/−^ mice that were treated with RA were grouped into sets of 3 (n = 4) and fed a chow, high-cholesterol, or high-CA diet for a further 2 days under continuous RA treatment; mRNA levels in the liver were then examined. Significant differences between the chow and special diet groups are indicated.

To examine gene expression during the acute phase with a biased diet, 12-week-old mice were fed either a high-cholesterol or high-CA diet for 2 days or a high-fat diet for 2 weeks. As shown in Figure 8A and S3A, the gene expression profile in the livers of young mice under chow-diet feeding was different from that of aged mice (Figure 3A), probably due to differences in the degree of hepatic injury (Figure 6A). Under the acute phase condition, the genes were categorized into 2 groups: (1) mRNA levels not affected by the diets in *Sik3* ^−/−^ mice (*e.g.*, *Shp*, *Bsep*, *AbcG5, AbcA1, and Fasn)*, and (2) mRNA levels that were irregularly affected (*e.g.*, *Cyp7a, Cyp8b,* and *Cyp27a)* (Figure 8A and S4A). Strangely, *Cyp7a* gene expression was lower in *Sik3* ^−/−^ mice than in wild-type mice, despite the low expression levels of *Shp*.

The little or no expression of *Cyp7a* observed under the high-cholesterol diet in *Sik3* ^−/−^ mice could be explained by LXR dysfunction [3], while the low expression of *Shp* or *Bsep* [6] and hypertrophic gallbladder [40] suggested FXR dysfunction. RXR is activated by 9-cis-RA, which is synthesized from vitamin A [9], and the impairment of RXR function affects vitamin A metabolism [42], resulting in the proliferation of bile duct epithelial cells (Figure 4B) [43]. Moreover, the livers of *Sik3* ^−/−^ mice expressed lower levels of *Rxr*αmRNA than the livers of wild-type mice, when the mice were fed with diets rich in cholesterol or CA (Figure S4B). Therefore, we decided to examine vitamin A metabolism in the livers of *Sik3* ^−/−^ mice by quantifying mRNA and protein levels.

The mRNA and protein levels of *cellular retinoid-binding protein 1* (*Crbp1*) and *retinal aldehyde dehydrogenase 1a* (*Aldh1a*) were up- and down-regulated, respectively, in *Sik3* ^−/−^ mice (Figure S4C and 8B). *Aldh1a* mRNA levels in the livers of *Sik3* ^−/−^ mice were also unaffected by the diet (Figure 8C). In addition, the livers of *Sik3* ^−/−^ mice contained higher levels of free retinol (vitamin A) than the livers of wild-type mice (Figure 8D). Moreover, treatment with 9-cis-RA rapidly reduced the levels of free retinol in the livers of *Sik3* ^−/−^ mice compared to the wild-type mice, suggesting that vitamin A metabolism might be impaired in *Sik3* ^−/−^ mice.

To further characterize these findings, the mice were treated with 9-cis-RA for 7 days, and several phenotypic parameters were then examined. Because 9-cis-RA is a pleiotropic compound, we first determined the minimum dose of 9-cis-RA as 4 mg kg^−1^·day^−1^ by monitoring the body weight and blood glucose levels of wild-type mice (Figure S5A and B). Treatment with 9-cis-RA induced weight gain in *Sik3* ^−/−^ mice (Figure 8E) and enabled them to maintain their blood glucose levels after fasting (Figure 8F). In addition, 9-cis-RA substantially decreased the levels of serum ALP and bile acid in *Sik3* ^−/−^ mice (Figure S5C). *Sik3* ^−/−^ mice treated with 9-cis-RA were also able to respond to nutritional stress by inducing the expression of metabolic markers (compare Figure 8G to 8A and Figure S5D). These results suggest that impaired vitamin A metabolism might be a cause of the phenotypes of *Sik3* ^−/−^ mice.

## Discussion

**Figure 9:**
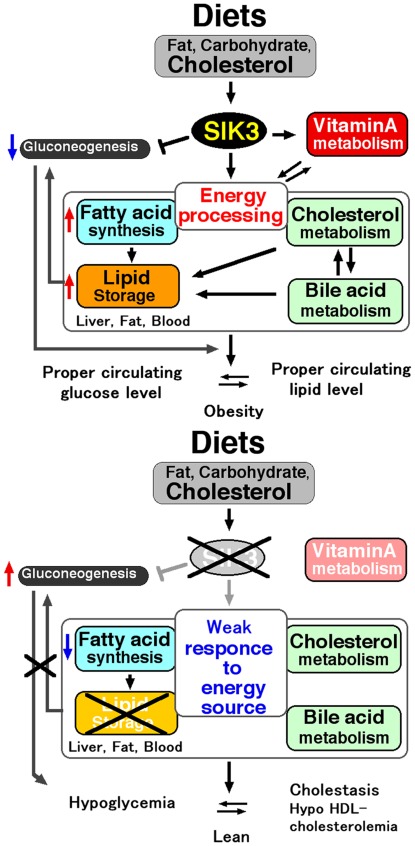
Summary of the metabolic events in wild-type (*upper*) and in *Sik3* ^−/−^ mice (*lower*).

Here, we have shown that SIK3 is induced in the liver when mice are fed a diet rich in fat, sucrose, and cholesterol. *Sik3* ^−/−^ mice present with a malnourished phenotype due to their reduced adaptation to excess nutrition, especially to cholesterol and CA, which eventually leads to severe cholestasis. These phenotypes are continuously observed even after 10 generations of cross-breeding with normal C57BL/6J mice, and we observed no substantial difference between males and females in their response to biased diets. Given these results, we propose that SIK3, in combination with vitamin A metabolism, is a novel regulator of cholesterol-BA homeostasis and lipid-storage size (Figure 9).

Previous studies suggested a direct contribution of RXR to cholesterol-BA homeostasis. Because the RXR ligand 9-cis-RA is synthesized from vitamin A, which is absorbed from enterocytes with the assistance of BA, its metabolism is tightly coupled to BA homeostasis [44]. A lack of vitamin A stimulates BA synthesis and its transport from hepatocytes to the bile ducts, *e.g*., via *Cyp7a* and *Bsep* gene expression [45], while excess 9-cis-RA inhibits their expression. The reduced expression of ALDH1a, an RA synthase, in the livers of *Sik3* ^−/−^ mice might be one of the causes of the *Sik3* ^−/−^ phenotype. All-trans-RA suppresses the expression of *Aldh1a* via an RAR-dependent mechanism [46], but 9-cis-RA does not, suggesting a distinct action for 9-cis-RA from all-trans-RA. Because physiological/endogenous 9-cis-RA has been identified only in the pancreas [47], [48], analyses of not only 9-cis-RA, but also its related substances in the liver are required to precisely characterize the *Sik3* ^−/−^ phenotype.

The administration of 9-cis-RA to *Sik3* ^−/−^ mice recovered the expression of *Cyp7a* and *Bsep* (Figure 7A and 7G), suggesting that the dose used (4 mg kg^−1^·d^−1^) may not be excessive for *Sik3* ^−/−^ mice. However, we have to mention that the wild-type mice that were fed with a vitamin A-deficient diet for 6 months from weaning did not develop cholestasis (unpublished observation), indicating that the levels of vitamin A and its metabolites may be insufficient to explain all of the *Sik3* ^−/−^ phenotypes. Meanwhile, free retinol (vitamin A) accumulated in the livers of *Sik3* ^−/−^ mice. Vitamin A toxicity is also suspected in hepatic cholestasis [49], suggesting that increased levels of free retinol may also contribute to the dysregulation of cholesterol-BA homeostasis in *Sik3* ^−/−^ mice. In addition, retinol aldehyde, a substrate of ALDH1a and a precursor of RA, is found to possess strong anti-obesity actions in mice [10].

Meanwhile, the high-fat diet ameliorated cholestasis in *Sik3* ^−/−^ mice (Figure 6A) without FA storage (in the liver and adipose tissues) or restoring the mRNA levels of genes involved in FA synthesis, such as *Fasn* (Figure 2 and Table S1). Interestingly, the high-fat diet up-regulated the expression of *thiolase*, a PPARα target, in the livers of *Sik3* ^−/−^ mice. PPARα is known to enhance bile flow [50] and some transcriptional pathways, such as *Shp* [51] (and also compare Table S1 to Figure 3A). Given that RXR is required for PPARα activation, its signaling may also be impaired in *Sik3* ^−/−^ mice, as expected from the gene expression profile observed for the chow diet (Figure 3A). These observations suggest that excess fat may stimulate a part of the downstream PPARα pathway (or close the gap downstream from PPARα that is impaired in *Sik3* ^−/−^ mice), which can improve BA homeostasis, probably in an SIK3-independent manner.

AMPK-related kinases are activated by the upstream kinase LKB1 [29], [52] SIK3 or AMPK–which kinase is important for the LKB1-mediated suppression of gluconeogenesis in the liver? Loss of LKB1 in the liver enhances the gluconeogenic program [53]. Since the gluconeogenic program in LKB-defective mice is resistant to the AMPK activator metformin, AMPK is proposed to be the kinase responsible for the LKB1-mediated regulation of gluconeogenesis. However, the livers of *Sik3* ^−/−^ mice possessed activated AMPK and an enhanced gluconeogenic program (Figure 3B), suggesting that loss of LKB1 causes a deficiency of SIK3 and subsequent AMPK resistance. In addition to gluconeogenesis, liver-specific LKB-defective mice present with severe cholestasis due to a lack of BSEP membrane-localization in the liver [54], while the apical side of the dilated canalicular structure was positive for BSEP in the livers of *Sik3* ^−/−^ mice, suggesting that the cause of cholestasis in these mice may not be identical to that in LKB-defective mice. We would again emphasize that the high-fat diet ameliorated cholestasis in *Sik3* ^−/−^ mice, indicating the importance of the process for nutrients rather than developmental defects in BA transportation.

Two recent reports have provided an argument against mechanisms by which SIK3 regulates energy balance. Mihalova *et al.* found that class 2a HDACs activated the FOXO transcription factor via deacetylation, thereby up-regulating gluconeogenesis in the liver [37]. Conversely, Wang *et al*. reported that loss of SIK2 in *Drosophila* (by disrupting the fly’s *Sik3* gene) resulted in the dephosphorylation of HDAC4 and the subsequent activation of FOXO [28]. Activated FOXO induces the lipolytic programs that reduce the lipid levels in body fat, thereby rendering the fly vulnerable to starvation. These data suggest that disinactivation of class 2a HDACs followed by the constitutive activation of FOXO may be a cause of the phenotypes of *Sik3* ^−/−^ mice.

Conversely, *Crebbp^+/−^* mice, with a mutant allele of the histone acetylase CREB-binding protein, displayed a phenotypes similar to that of *Sik3^−/−^* mice, *e.g.*, lipodystrophy, increased glucose tolerance, resistance to diet-induced obesity, and hyperadiponectinemia [55], suggesting that SIK3 may regulate energy balance by regulating acetylation states. The levels of adipose tissue and circulating blood lipids may be important for buffering cholesterol and protecting the liver from cholesterol toxicity, which in turn, increases the risk of obesity and hyperlipidemia (Figure 9).

In the present study, profiling the metabolic changes in *Sik3* ^−/−^ mice represents a new start to the study SIK and may also provide novel insights into the metabolic diseases caused by Western diets. Another remarkable phenotype of *Sik3* ^−/−^ mice is found in the differentiation of chondrocytes [31], and a number of interactions between energy metabolism and skeletal development have been reported, *e.g.,* insulin [56], leptin [57], adiponectin [58], osteocalcin [59], [60], and inflammatory cytokines [61]. Further analyses of the cell autonomous functions of SIK3 and of systemic or developmental abnormalities in the organs for energy metabolicsm in *Sik3* ^−/−^ mice are needed.

## Materials and Methods

### Sik3^−/−^ Mice

Embryonic stem cells derived from a C57BL/6N strain (RENKA) were used with the *Sik3^−/−^* mice. After mating the mice with C57BL/6J mice (CLEA Japan, Tokyo, Japan) for 3 generations, mouse colonies were expanded for experiments under chow and high-fat-diet feeding. After 7 generations of cross breeding, mice colonies were used for cholesterol and cholic acid experiments. *Sik3^+/−^* mice are now supplied by JCRB Laboratory Animal Resource Bank at the National Institute of Biomedical Innovation (No. nbio157). The experimental mouse protocols were approved by the ethics committee at the National Institute of Biomedical Innovation (assigned No. DS-20-56). The animals were maintained under standard conditions of light (0800–2000) and temperature (23°C, 50% humidity).

For tissue isolation, all mice were fasted for 4 h and then sacrificed within ±1 h of lights out. The chow diet, MF, was purchased from Oriental Yeast (Tokyo, Japan). The high-sucrose (20% cal), high-fat (60% cal), and high-fat (45% cal)/high-sucrose (20% cal) diets were obtained from Research DIET Inc. (NJ, USA). We supplemented 2% cholesterol in the high-fat and high-fat/high-sucrose diets. To prepare the high-cholesterol and high-cholic acid (CA) diets, the chow diet was supplemented with 2% cholesterol or 0.25% CA, respectively. O_2_ consumption was monitored using the Oxymax system (Columbus Instruments, Columbus, OH, USA).

The pre-fasting periods for the glucose tolerance test (GTT), insulin tolerance test (ITT), and lactate tolerance test (LTT) were 4, 2, and 24 h, respectively. We administered 1.5 g/kg glucose, 36 µg/kg insulin, and 1.5 g/kg lactate intraperitoneally for these tests, respectively.

### Fractionation of Hepatic Parenchymal Cells and Non-parenchymal Cells

Under anesthesia by isoflurane, female C57BL/6J mice (12-week-old) were perfused with Hank’s balanced salt solution containing 0.5 mM EGTA via inferior vena cava followed by perfusion with Liver Digestion Medium (Invitorgen). After the digestion, hepatic cells were suspended in Dulbecco’s Modified Eagle Medium (DMEM) supplemented with 10% fetal bovine serum and centrifuged at 40×g for 2 minutes. The pellet was used for the parenchymal-cell fraction, and the supernatant was recovered by further centrifuged at 800×g for 5 min and used for non-parenchymal-cell fraction.

### Reagents

Blood glucose and β-hydroxybutyrate were measured using a G-meter (Arkray, Kyoto, Japan) and Precision Xceed (Abbott, Abbott Park, IL, USA), respectively. Total cholesterol and triglyceride (TG) in sera were measured using a DryChem7000 (Fujifilm, Tokyo, Japan). Lipids in the liver or feces were extracted in 10 volumes of methanol:chloroform (1∶2), dried under N_2_ gas, suspended in 300 µL t-butyl alcohol:methanol:Triton-X 100 (2∶1∶1, v/v), and quantified using kits (WAKO, Osaka, Japan). Serum insulin, leptin and adiponectin levels were measured using enzyme-linked immunosorbent assay kits from Shibayagi (Gunma, Japan), and low levels of insulin were measured with a low range kit from Morinaga (Tokyo, Japan), while free FA and BA levels were measured using kits from WAKO. Serum lipid separation by fast protein liquid chromatography (FPLC) was contracted to LipiSEARCH (Skylight Biotech, Akita, Japan). The anti-AMPK, anti-phospho-AMPK, and anti-HDAC5 antibodies were purchased from Cell Signaling (Boston, MA, USA), anti-BSEP antibody were from ABGENT (San Diego, CA, USA), while the anti-ALDH1a and anti-CRBP1 antibodies were obtained from Epitomics (Burlingame, CA, USA). The anti-CRTC2 antibody was described previously [29].

### Quantitative Real-time PCR

Total RNA was extracted using an EZ1 RNA Universal Tissue Kit (Qiagen, Venlo Park, Netherlands), and cDNA was synthesized using a Transcriptor cDNA First Strand Synthesis Kit (Roche, Branford, CT, USA). PCR amplification was performed using Platinum Quantitative PCR SuperMix (Invitrogen). Since the level of the internal standard RNA, 36B4, was induced by the CA-rich diet, the expression levels of mRNA in the liver of mice fed with diets supplemented with CA or Chol were normalized using glyceraldehyde 3-phosphate dehydrogenase levels. Gene names, abbreviations and primer sequence used in the quantities PCR analysis are listed in Table S2.

### Statistical Analysis

Student’s *t*-test was used to assess all experimental data in Microsoft Excel. The mean and standard error of the mean (SEM) are shown.

## Supporting Information

Figure S1 (A) Most *Sik3* ^−/−^ mice died on the first day after birth. The mating system and time of genotyping are indicated. The percentage and number of mice in the first column indicate the sum of neonates at day 1 and embryos at E17.5–E18.5. Neonates prepared by *in vitro* fertilization were delivered by cesarean section and living mice were counted without genotyping. However, ∼50% of the mice disappeared by the second day, probably because they were eaten by the foster mice. (B) The difference in the body size of *Sik3* ^−/−^ mice became obvious after 2 weeks. (C) HE staining of gonadal fat of 1-year-old mice. (D) Cholesterol (Chol), triglyceride (TG), and carbohydrate (Carbo) content in feces (from 3 cages). Cholesterol and triglycerides were extracted with methanol/chloroform as described in the Materials and Methods. To extract undigested carbohydrates, the feces were re-digested with amylase at 37°C for 12 h, and the debris was removed by centrifugation. Carbohydrates were stained with a solution of 1 volume of 5% phenol and 5 volumes of sulfuric acid and then detected at 490 nm. (E) After fasting for 4-h fasting, the serum levels of free thyroid hormones (FT3 and FT4) were measured with an automated system for clinical assays. Serum thyroid stimulating hormone (TSH) levels were measured with an ELISA kit from Shibayagi Co., Ltd. (F) Insulin tolerance test (ITT). Mice (male n = 5) were fasted for 2 h and then treated intraperitoneally with 36 µg/kg insulin. All data points are *p*<0.001.
(TIF)

Figure S2 (A) Body weight curves of wild-type and *Sik3* heterozygous mice are also shown (n  = 12). (B) Levels of *Sik3* mRNA in the livers, brown adipose tissues (BAT), and muscles of wild-type, heterozygous, and *Sik3* ^−/−^ mice (n = 3). The error bars indicate SEM. Levels of SIK3 protein in the livers of wild-type, heterozygous, and *Sik3* ^−/−^ mice. (C) Hepatic parenchymal and non-parenchymal cells were separated by centrifugation, and *Sik3* mRNA levels were examined by quantitative PCR. *Cyp7α*, *F4/80*, and *Desmin* were used as markers for parenchymal cells, Kupffer’s cells (non-parenchymal), and hepatic stellate cells (non-parenchymal), respectively. (n = 3: means and SEM are shown). (D) *In vitro* adipocyte differentiation assay. Preadipocytes were prepared from gonadal fat pads using collagenase and then plated. When the cells reached confluence, the culture medium was changed to Dulbecco’s Modified Eagle’s Medium (high glucose) supplemented with rosiglitazone (Rosi: indicated concentration), and insulin (1 µg/mL). After 8 days (with changes of medium every 2 days), the cells were fixed with 4% paraformaldehyde and stained with Oil Red O. The high magnification images show cells that were differentiated using 3 µM rosiglitazone. (F) Serum adiponectin levels of the mice examined in Figure 3E. Means and SEM are shown. ### indicates *p*<0.001.
(TIF)

Figure S3 (A) HE staining of embryo livers. The sets in the left and right panels are the same magnification. The lower panels are a higher magnification of the upper panels. (B) Bile acid was extracted with 95% ethanol/0.5% NH_3_-water. The numbers of mice (wild-type and *Sik3^−/−^*) used for the assay were: E16.5, 11 and 6; E18.5, 16 and 3; P0, 9 and 5; and 12 weeks, 8 and 5, respectively. Means and SEM are shown. Significant differences between wild-type and *Sik3* ^−/−^ mice are shown by * for *p*<0.05. ## indicates significant differences between P0 and E18.5 or 12 weeks in wild-type mice (*p*<0.01).
(TIF)

Figure S4 (A) Male mice (12 weeks of age, n = 3) were fed diets supplemented with Chol (2%) and cholic acid (0.25%) for 2 days or with fat (60% of calories) for 2 weeks and then sacrificed. The expression of genes for Chol and BA metabolism in the liver was examined using quantitative polymerase chain reaction (normalized by glyceraldehyde 3-phosphate dehydrogenase [GAPDH] levels). Significant differences between wild-type and *Sik3* ^−/−^ mice are shown by *, **, and *** for *p*<0.05, <0.01, and <0.001, respectively. # indicates a significant difference between the chow and special diet groups. Means and SEM are shown. (B) Expression levels of nuclear receptors. (C) The expression of genes involved in vitamin A metabolism was examined using the liver cDNA in Figure 3A (1-year-old mice, n = 5).
(TIF)

Figure S5 (A) Effect of 9-cis-RA treatment (0–16 mg kg^−1^·d^−1^) on the weight gain of wild-type mice (n = 6). (B) Blood glucose levels before and after treatment are indicated by labels as B and A, respectively. (C) The levels of serum ALP and bile acids were measured before (labeled as B) and after (labeled as A) 9-cis-RA treatment (for 9 days: after the analysis shown in Figure 8E). Ethanol (EtOH, 1%) was used as a solvent. Significant differences before and after treatment in the same group (n = 4) are indicated. Although there were no significant fluctuations in the levels of bile acids, their levels decreased in all *Sik3* ^−/−^ mice after treatment. (D) Effect of 9-cis-RA on gene expression in *Sik3^−/−^* mice. At day 7, *Sik3* ^−/−^ mice treated with 9-cis-RA were grouped into sets of 3 (n = 4) and fed a chow, high-Chol, or high-CA diet for an additional 2 days under continuous RA treatment; mRNA levels in the liver were then examined. Significant differences between the chow and special diet groups are indicated.
(TIF)

Table S1 mRNA levels in the liver.
(XLS)

Table S2 List of primers used for quantitative-PCR.
(XLS)

## References

[1] Wagner M, Zollner G, Trauner M (2011). Nuclear receptors in liver disease.. Hepatology.

[2] Willy PJ, Umesono K, Ong ES, Evans RM, Heyman RA (1995). LXR, a nuclear receptor that defines a distinct retinoid response pathway.. Genes Dev.

[3] Peet DJ, Turley SD, Ma W, Janowski BA, Lobaccaro JM (1998). Cholesterol and bile acid metabolism are impaired in mice lacking the nuclear oxysterol receptor LXR alpha.. Cell.

[4] Kalaany NY, Gauthier KC, Zavacki AM, Mammen PP, Kitazume T (2005). LXRs regulate the balance between fat storage and oxidation.. Cell Metab.

[5] Makishima M, Okamoto AY, Repa JJ, Tu H, Learned RM (1999). Identification of a nuclear receptor for bile acids.. Science.

[6] Sinal CJ, Tohkin M, Miyata M, Ward JM, Lambert G (2000). Targeted disruption of the nuclear receptor FXR/BAR impairs bile acid and lipid homeostasis.. Cell.

[7] Watanabe M, Houten SM, Mataki C, Christoffolete MA, Kim BW (2006). Bile acids induce energy expenditure by promoting intracellular thyroid hormone activation.. Nature.

[8] Watanabe M, Horai Y, Houten SM, Morimoto K, Sugizaki T (2011). Lowering bile acid pool size with a synthetic farnesoid X receptor (FXR) agonist induces obesity and diabetes through reduced energy expenditure.. J Biol Chem.

[9] Heyman RA, Mangelsdorf DJ, Dyck JA, Stein RB, Eichele G (1992). 9-cis retinoic acid is a high affinity ligand for the retinoid X receptor.. Cell.

[10] Ziouzenkova O, Orasanu G, Sharlach M, Akiyama TE, Berger JP (2007). Retinaldehyde represses adipogenesis and diet-induced obesity.. Nat Med.

[11] Iqbal J, Hussain MM (2009). Intestinal lipid absorption.. Am J Physiol Endocrinol Metab.

[12] Takemori H, Okamoto M (2008). Regulation of CREB-mediated gene expression by salt inducible kinase.. J Steroid Biochem Mol Biol.

[13] Doi J, Takemori H, Lin X-z, Horike N, Katoh Y (2002). Salt-inducible kinase represses PKA-mediated activation of human cholesterol side chain cleavage cytochrome promoter through the CREB basic leucine zipper domain.. J Biol Chem.

[14] Takemori H, Katoh Y, Horike N, Doi J, Okamoto M (2002). ACTH-induced nucleocytoplasmic translocation of salt-inducible kinase. Implication in the protein kinase A-activated gene transcription in mouse adrenocortical tumor cells.. J Biol Chem.

[15] Koo SH, Flechner L, Qi L, Zhang X, Screaton RA (2005). The CREB Coactivator TORC2 is a Key Regulator of Fasting Glucose Metabolism.. Nature.

[16] Uebi T, Tamura M, Horike N, Hashimoto YK, Takemori H (2010). Phosphorylation of the CREB-specific coactivator TORC2 at Ser(307) regulates its intracellular localization in COS-7 cells and in the mouse liver.. Am J Physiol Endocrinol Metab.

[17] Muraoka M, Fukushima A, Viengchareun S, Lombes M, Kishi F (2009). Involvement of SIK2/TORC2 signaling cascade in the regulation of insulin-induced PGC-1alpha and UCP-1 gene expression in brown adipocytes.. Am J Physiol Endocrinol Metab.

[18] Sasaki T, Takemori H, Yagita Y, Terasaki Y, Uebi T (2011). SIK2 is a key regulator for neuronal survival after ischemia via TORC1-CREB.. Neuron.

[19] Horike N, Kumagai A, Shimono Y, Onishi T, Itoh Y (2010). Downregulation of SIK2 expression promotes the melanogenic program in mice.. Pigment Cell Melanoma Res.

[20] Kumagai A, Horike N, Satoh Y, Uebi T, Sasaki T (2011). A Potent Inhibitor of SIK2, 3, 3′, 7-Trihydroxy-4′-Methoxyflavon (4′-O-Methylfisetin), Promotes Melanogenesis in B16F10 Melanoma Cells.. PLoS One.

[21] Altarejos JY, Montminy M (2011). CREB and the CRTC co-activators: sensors for hormonal and metabolic signals.. Nat Rev Mol Cell Biol.

[22] Conkright MD, Canettieri G, Screaton R, Guzman E, Miraglia L (2003). TORCs: transducers of regulated CREB activity.. Mol Cell.

[23] Iourgenko V, Zhang W, Mickanin C, Daly I, Jiang C (2003). Identification of a family of cAMP response element-binding protein coactivators by genome-scale functional analysis in mammalian cells.. Proc Natl Acad Sci U S A.

[24] Screaton RA, Conkright MD, Katoh Y, Best JL, Canettieri G (2004). The CREB coactivator TORC2 functions as a calcium- and cAMP-sensitive coincidence detector.. Cell.

[25] Katoh Y, Takemori H, Min L, Muraoka M, Doi J (2004). Salt-inducible kinase-1 represses cAMP response element-binding protein activity both in the nucleus and in the cytoplasm.. Eur J Biochem.

[26] Bricambert J, Miranda J, Benhamed F, Girard J, Postic C (2010). Salt-inducible kinase 2 links transcriptional coactivator p300 phosphorylation to the prevention of ChREBP-dependent hepatic steatosis in mice.. J Clin Invest.

[27] Berdeaux R, Goebel N, Banaszynski L, Takemori H, Wandless T (2007). SIK1 is a class II HDAC kinase that promotes survival of skeletal myocytes.. Nat Med.

[28] Wang B, Moya N, Niessen S, Hoover H, Mihaylova MM (2011). A hormone-dependent module regulating energy balance.. Cell.

[29] Katoh Y, Takemori H, Lin XZ, Tamura M, Muraoka M (2006). Silencing the constitutive active transcription factor CREB by the LKB1-SIK signaling cascade.. Febs J.

[30] Hashimoto YK, Satoh T, Okamoto M, Takemori H (2008). Importance of autophosphorylation at Ser186 in the A-loop of salt inducible kinase 1 for its sustained kinase activity.. J Cell Biochem.

[31] Sasagawa S, Takemori H, Uebi T, Ikegami D, Hiramatsu K (2012). SIK3 is essential for chondrocyte hypertrophy during skeletal development in mice.. Development.

[32] Lanjuin A, Sengupta P (2002). Regulation of chemosensory receptor expression and sensory signaling by the KIN-29 Ser/Thr kinase.. Neuron.

[33] Wang B, Goode J, Best J, Meltzer J, Schilman PE (2008). The insulin-regulated CREB coactivator TORC promotes stress resistance in Drosophila.. Cell Metab.

[34] Hurov JB, Huang M, White LS, Lennerz J, Choi CS (2007). Loss of the Par-1b/MARK2 polarity kinase leads to increased metabolic rate, decreased adiposity, and insulin hypersensitivity in vivo.. Proc Natl Acad Sci U S A.

[35] Lennerz JK, Hurov JB, White LS, Lewandowski KT, Prior JL (2010). Loss of Par-1a/MARK3/C-TAK1 kinase leads to reduced adiposity, resistance to hepatic steatosis, and defective gluconeogenesis.. Mol Cell Biol.

[36] Inagaki T, Dutchak P, Zhao G, Ding X, Gautron L (2007). Endocrine regulation of the fasting response by PPARalpha-mediated induction of fibroblast growth factor 21.. Cell Metab.

[37] Mihaylova MM, Vasquez DS, Ravnskjaer K, Denechaud PD, Yu RT (2011). Class IIa Histone Deacetylases Are Hormone-Activated Regulators of FOXO and Mammalian Glucose Homeostasis.. Cell.

[38] Takemori H, Katoh-Hashimoto Y, Nakae J, Olson EN, Okamoto M (2009). Inactivation of HDAC5 by SIK1 in AICAR-treated C2C12 myoblasts.. Endocr J.

[39] Kim JY, van de Wall E, Laplante M, Azzara A, Trujillo ME (2007). Obesity-associated improvements in metabolic profile through expansion of adipose tissue.. J Clin Invest.

[40] Moschetta A, Bookout AL, Mangelsdorf DJ (2004). Prevention of cholesterol gallstone disease by FXR agonists in a mouse model.. Nat Med.

[41] Moschetta A, vanBerge-Henegouwen GP, Portincasa P, Renooij WL, Groen AK (2001). Hydrophilic bile salts enhance differential distribution of sphingomyelin and phosphatidylcholine between micellar and vesicular phases: potential implications for their effects in vivo.. J Hepatol.

[42] Gyamfi MA, He L, French SW, Damjanov I, Wan YJ (2008). Hepatocyte retinoid X receptor alpha-dependent regulation of lipid homeostasis and inflammatory cytokine expression contributes to alcohol-induced liver injury.. J Pharmacol Exp Ther.

[43] Weiss B, Barshack I, Onaca N, Goldberg I, Berkovich Z (2010). Vitamin A deficiency associated with enhanced proliferation of bile duct epithelial cells in the rat.. Isr Med Assoc J.

[44] Schmidt DR, Holmstrom SR, Fon Tacer K, Bookout AL, Kliewer SA (2010). Regulation of bile acid synthesis by fat-soluble vitamins A and D. J Biol Chem.

[45] Hoeke MO, Plass JR, Heegsma J, Geuken M, van Rijsbergen D (2009). Low retinol levels differentially modulate bile salt-induced expression of human and mouse hepatic bile salt transporters.. Hepatology.

[46] Elizondo G, Corchero J, Sterneck E, Gonzalez FJ (2000). Feedback inhibition of the retinaldehyde dehydrogenase gene ALDH1 by retinoic acid through retinoic acid receptor alpha and CCAAT/enhancer-binding protein beta.. J Biol Chem.

[47] Kane MA, Folias AE, Pingitore A, Perri M, Obrochta KM (2010). Identification of 9-cis-retinoic acid as a pancreas-specific autacoid that attenuates glucose-stimulated insulin secretion.. Proc Natl Acad Sci U S A.

[48] Kane MA (2012). Analysis, occurrence, and function of 9-cis-retinoic acid..

[49] Erickson JM, Mawson AR (2000). Possible role of endogenous retinoid (Vitamin A) toxicity in the pathophysiology of primary biliary cirrhosis.. J Theor Biol.

[50] Kok T, Bloks VW, Wolters H, Havinga R, Jansen PL (2003). Peroxisome proliferator-activated receptor alpha (PPARalpha)-mediated regulation of multidrug resistance 2 (Mdr2) expression and function in mice.. Biochem J.

[51] Chanda D, Lee CH, Kim YH, Noh JR, Kim DK (2009). Fenofibrate differentially regulates plasminogen activator inhibitor-1 gene expression via adenosine monophosphate-activated protein kinase-dependent induction of orphan nuclear receptor small heterodimer partner.. Hepatology.

[52] Lizcano JM, Goransson O, Toth R, Deak M, Morrice NA (2004). LKB1 is a master kinase that activates 13 kinases of the AMPK subfamily, including MARK/PAR-1.. Embo J.

[53] Shaw RJ, Lamia KA, Vasquez D, Koo SH, Bardeesy N (2005). The kinase LKB1 mediates glucose homeostasis in liver and therapeutic effects of metformin.. Science.

[54] Woods A, Heslegrave AJ, Muckett PJ, Levene AP, Clements M (2011). LKB1 is required for hepatic bile acid transport and canalicular membrane integrity in mice.. Biochem J.

[55] Yamauchi T, Kamon J, Minokoshi Y, Ito Y, Waki H (2002). Adiponectin stimulates glucose utilization and fatty-acid oxidation by activating AMP-activated protein kinase.. Nat Med.

[56] Wu S, Aguilar AL, Ostrow V, De Luca F (2011). Insulin resistance secondary to a high-fat diet stimulates longitudinal bone growth and growth plate chondrogenesis in mice.. Endocrinology.

[57] Yadav VK, Oury F, Suda N, Liu ZW, Gao XB (2009). A serotonin-dependent mechanism explains the leptin regulation of bone mass, appetite, and energy expenditure.. Cell.

[58] Maeda T, Jikko A, Abe M, Yokohama-Tamaki T, Akiyama H (2006). Cartducin, a paralog of Acrp30/adiponectin, is induced during chondrogenic differentiation and promotes proliferation of chondrogenic precursors and chondrocytes.. J Cell Physiol.

[59] Ferron M, Wei J, Yoshizawa T, Del Fattore A, DePinho RA (2010). Insulin signaling in osteoblasts integrates bone remodeling and energy metabolism.. Cell.

[60] Fulzele K, Riddle RC, DiGirolamo DJ, Cao X, Wan C (2010). Insulin receptor signaling in osteoblasts regulates postnatal bone acquisition and body composition.. Cell.

[61] Shikhman AR, Brinson DC, Valbracht J, Lotz MK (2001). Cytokine regulation of facilitated glucose transport in human articular chondrocytes.. J Immunol.

