# Supplementary figures and images for: Involvement of SIK3 in Glucose and Lipid Homeostasis in Mice
Source: PLoS One. 2012 May 25;7(5):e37803. doi: 10.1371/journal.pone.0037803 (PMC3360605; doi:10.1371/journal.pone.0037803)

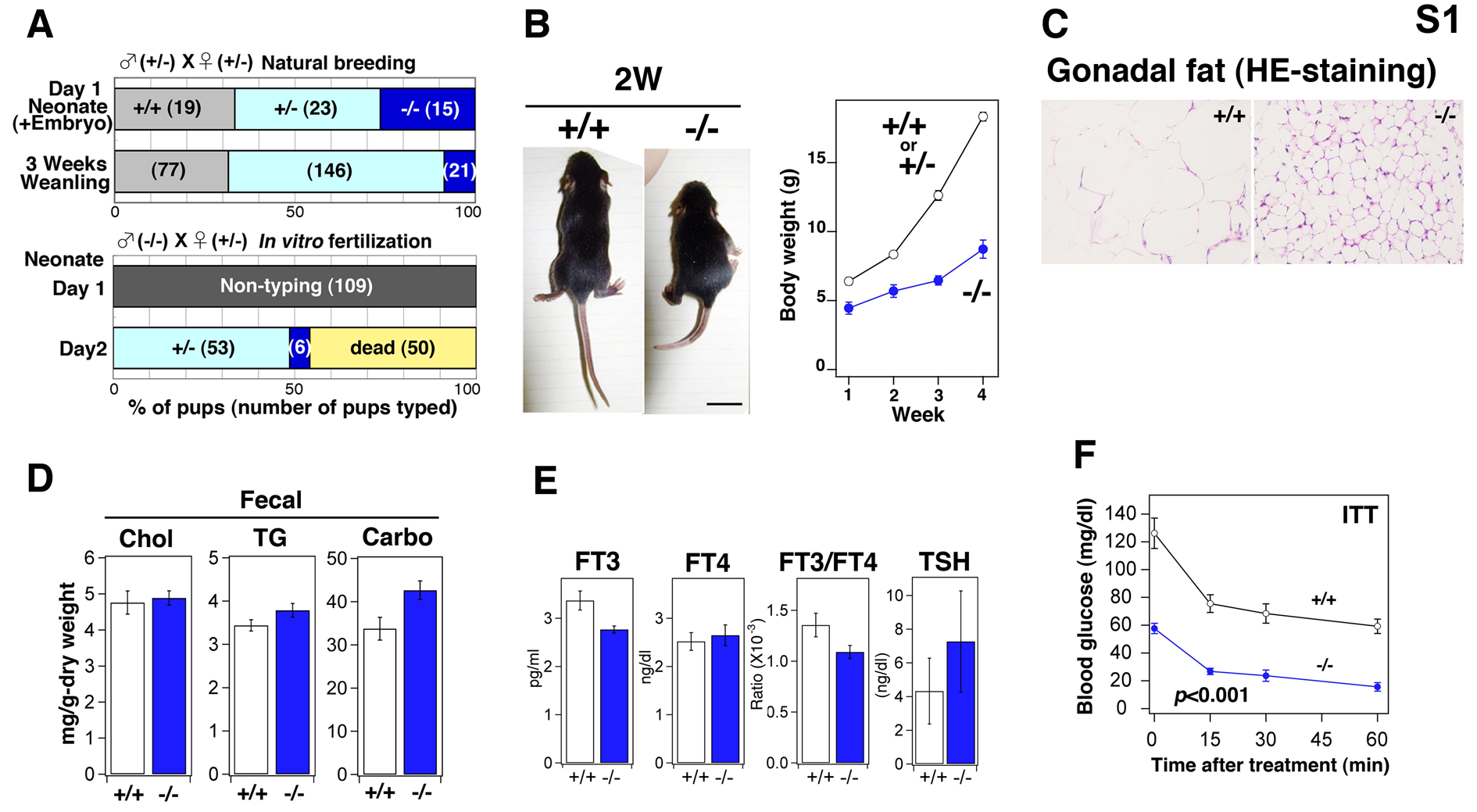

Supplement: Figure S1 — (A) Most Sik3 −/− mice died on the first day after birth. The mating system and time of genotyping are indicated. The percentage and number of mice in the first column indicate the sum of neonates at day 1 and embryos at E17.5–E18.5. Neonates prepared by in vitro fertilization were delivered by cesarean section and living mice were counted without genotyping. However, ∼50% of the mice disappeared by the second day, probably because they were eaten by the foster mice. (B) The difference in the body size of Sik3 −/− mice became obvious after 2 weeks. (C) HE staining of gonadal fat of 1-year-old mice. (D) Cholesterol (Chol), triglyceride (TG), and carbohydrate (Carbo) content in feces (from 3 cages). Cholesterol and triglycerides were extracted with methanol/chloroform as described in the Materials and Methods. To extract undigested carbohydrates, the feces were re-digested with amylase at 37°C for 12 h, and the debris was removed by centrifugation. Carbohydrates were stained with a solution of 1 volume of 5% phenol and 5 volumes of sulfuric acid and then detected at 490 nm. (E) After fasting for 4-h fasting, the serum levels of free thyroid hormones (FT3 and FT4) were measured with an automated system for clinical assays. Serum thyroid stimulating hormone (TSH) levels were measured with an ELISA kit from Shibayagi Co., Ltd. (F) Insulin tolerance test (ITT). Mice (male n = 5) were fasted for 2 h and then treated intraperitoneally with 36 µg/kg insulin. All data points are p<0.001. (TIF) [file pone.0037803.s001.tif]

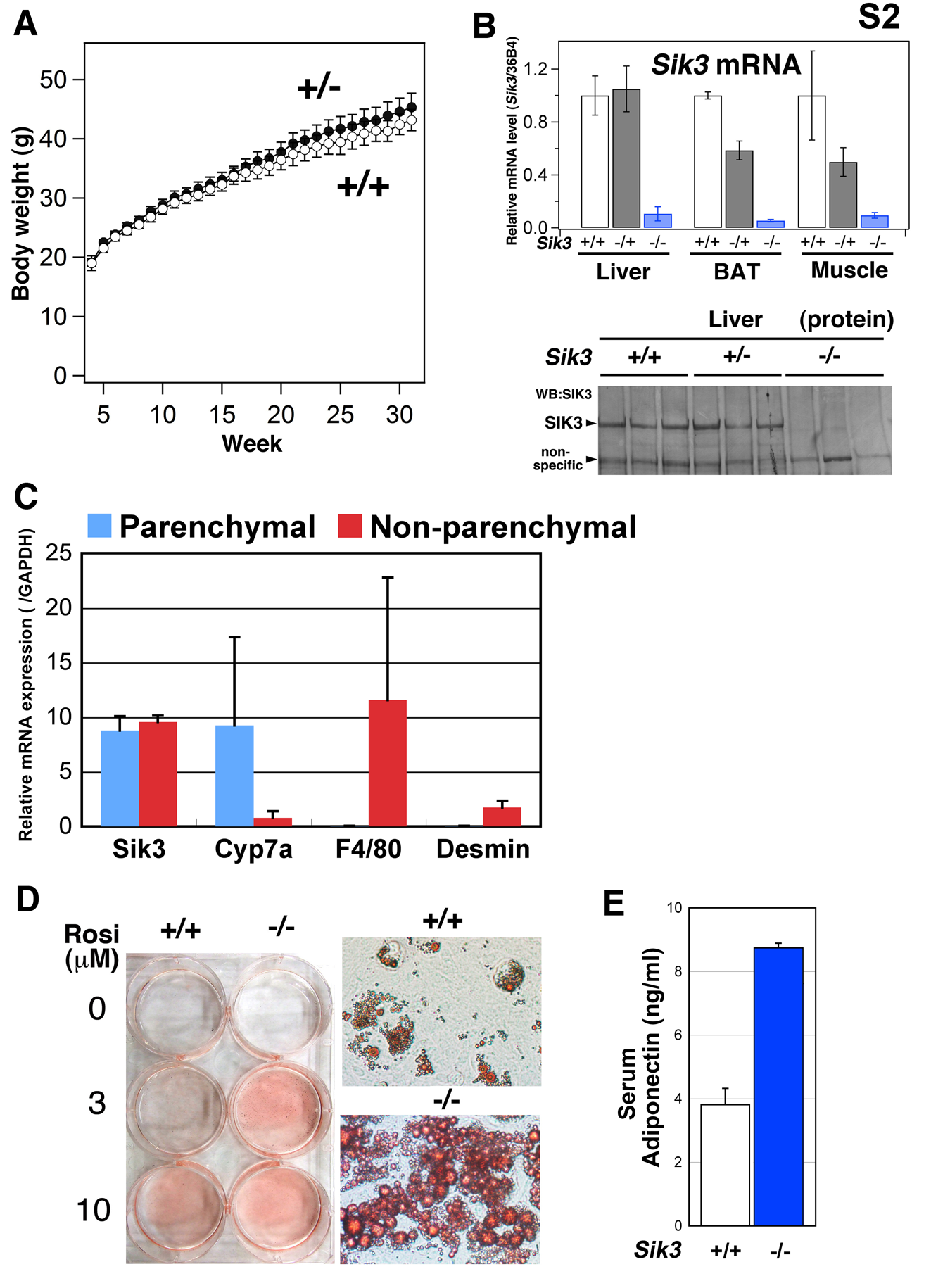

Supplement: Figure S2 — (A) Body weight curves of wild-type and Sik3 heterozygous mice are also shown (n = 12). (B) Levels of Sik3 mRNA in the livers, brown adipose tissues (BAT), and muscles of wild-type, heterozygous, and Sik3 −/− mice (n = 3). The error bars indicate SEM. Levels of SIK3 protein in the livers of wild-type, heterozygous, and Sik3 −/− mice. (C) Hepatic parenchymal and non-parenchymal cells were separated by centrifugation, and Sik3 mRNA levels were examined by quantitative PCR. Cyp7α, F4/80, and Desmin were used as markers for parenchymal cells, Kupffer’s cells (non-parenchymal), and hepatic stellate cells (non-parenchymal), respectively. (n = 3: means and SEM are shown). (D) In vitro adipocyte differentiation assay. Preadipocytes were prepared from gonadal fat pads using collagenase and then plated. When the cells reached confluence, the culture medium was changed to Dulbecco’s Modified Eagle’s Medium (high glucose) supplemented with rosiglitazone (Rosi: indicated concentration), and insulin (1 µg/mL). After 8 days (with changes of medium every 2 days), the cells were fixed with 4% paraformaldehyde and stained with Oil Red O. The high magnification images show cells that were differentiated using 3 µM rosiglitazone. (F) Serum adiponectin levels of the mice examined in Figure 3E. Means and SEM are shown. ### indicates p<0.001. (TIF) [file pone.0037803.s002.tif]

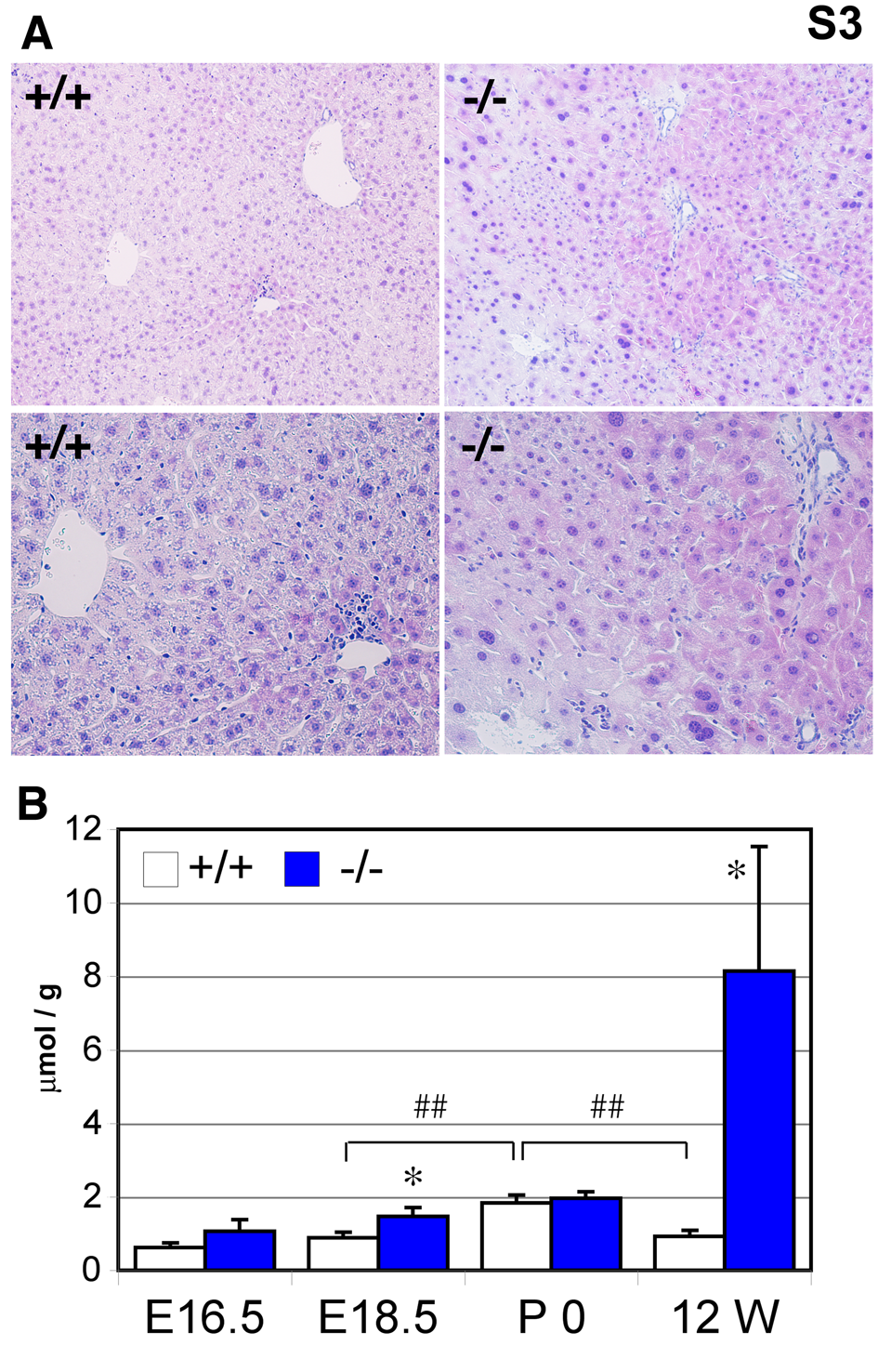

Supplement: Figure S3 — (A) HE staining of embryo livers. The sets in the left and right panels are the same magnification. The lower panels are a higher magnification of the upper panels. (B) Bile acid was extracted with 95% ethanol/0.5% NH3-water. The numbers of mice (wild-type and Sik3−/−) used for the assay were: E16.5, 11 and 6; E18.5, 16 and 3; P0, 9 and 5; and 12 weeks, 8 and 5, respectively. Means and SEM are shown. Significant differences between wild-type and Sik3 −/− mice are shown by * for p<0.05. ## indicates significant differences between P0 and E18.5 or 12 weeks in wild-type mice (p<0.01). (TIF) [file pone.0037803.s003.tif]

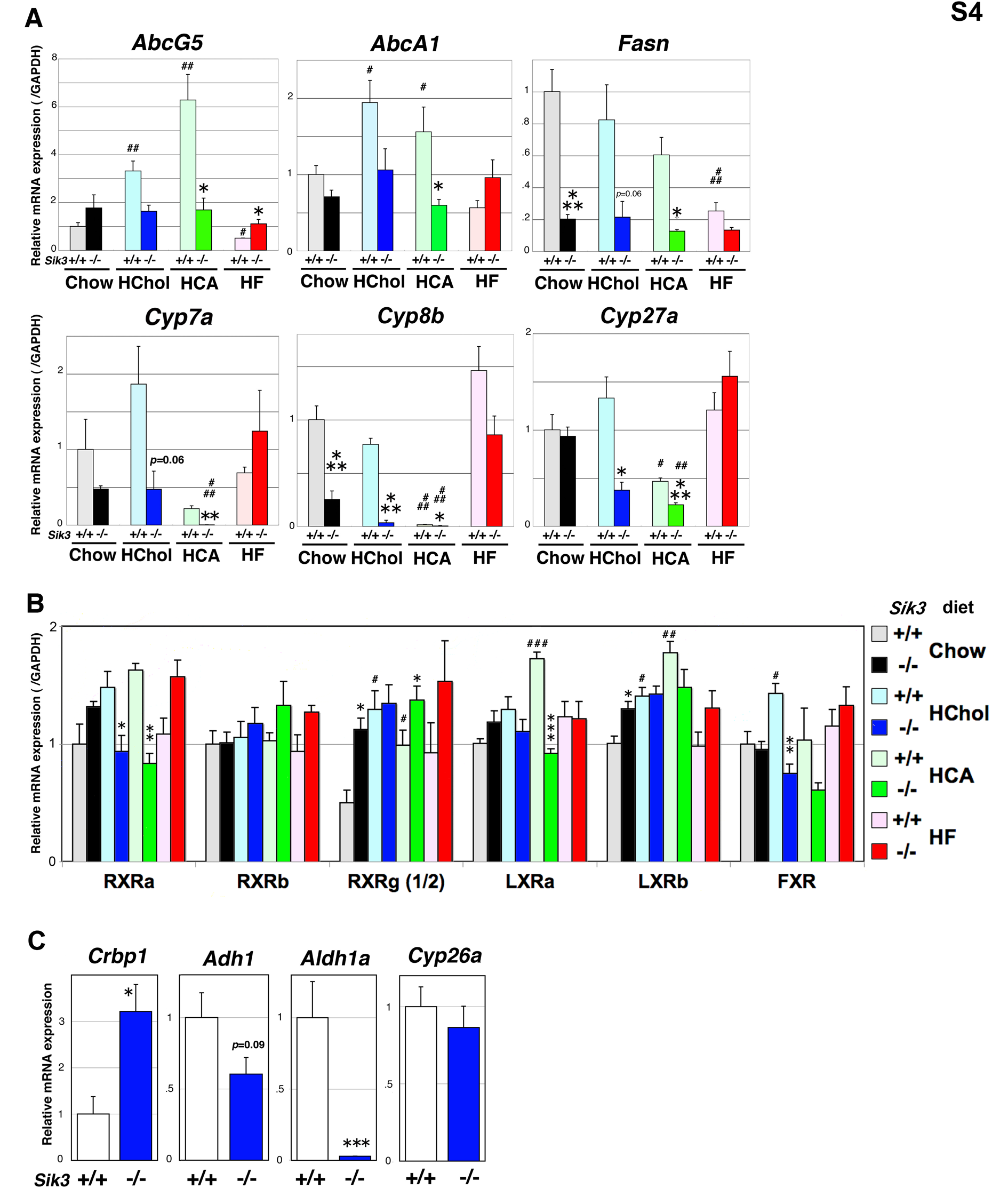

Supplement: Figure S4 — (A) Male mice (12 weeks of age, n = 3) were fed diets supplemented with Chol (2%) and cholic acid (0.25%) for 2 days or with fat (60% of calories) for 2 weeks and then sacrificed. The expression of genes for Chol and BA metabolism in the liver was examined using quantitative polymerase chain reaction (normalized by glyceraldehyde 3-phosphate dehydrogenase [GAPDH] levels). Significant differences between wild-type and Sik3 −/− mice are shown by *, **, and *** for p<0.05, <0.01, and <0.001, respectively. # indicates a significant difference between the chow and special diet groups. Means and SEM are shown. (B) Expression levels of nuclear receptors. (C) The expression of genes involved in vitamin A metabolism was examined using the liver cDNA in Figure 3A (1-year-old mice, n = 5). (TIF) [file pone.0037803.s004.tif]

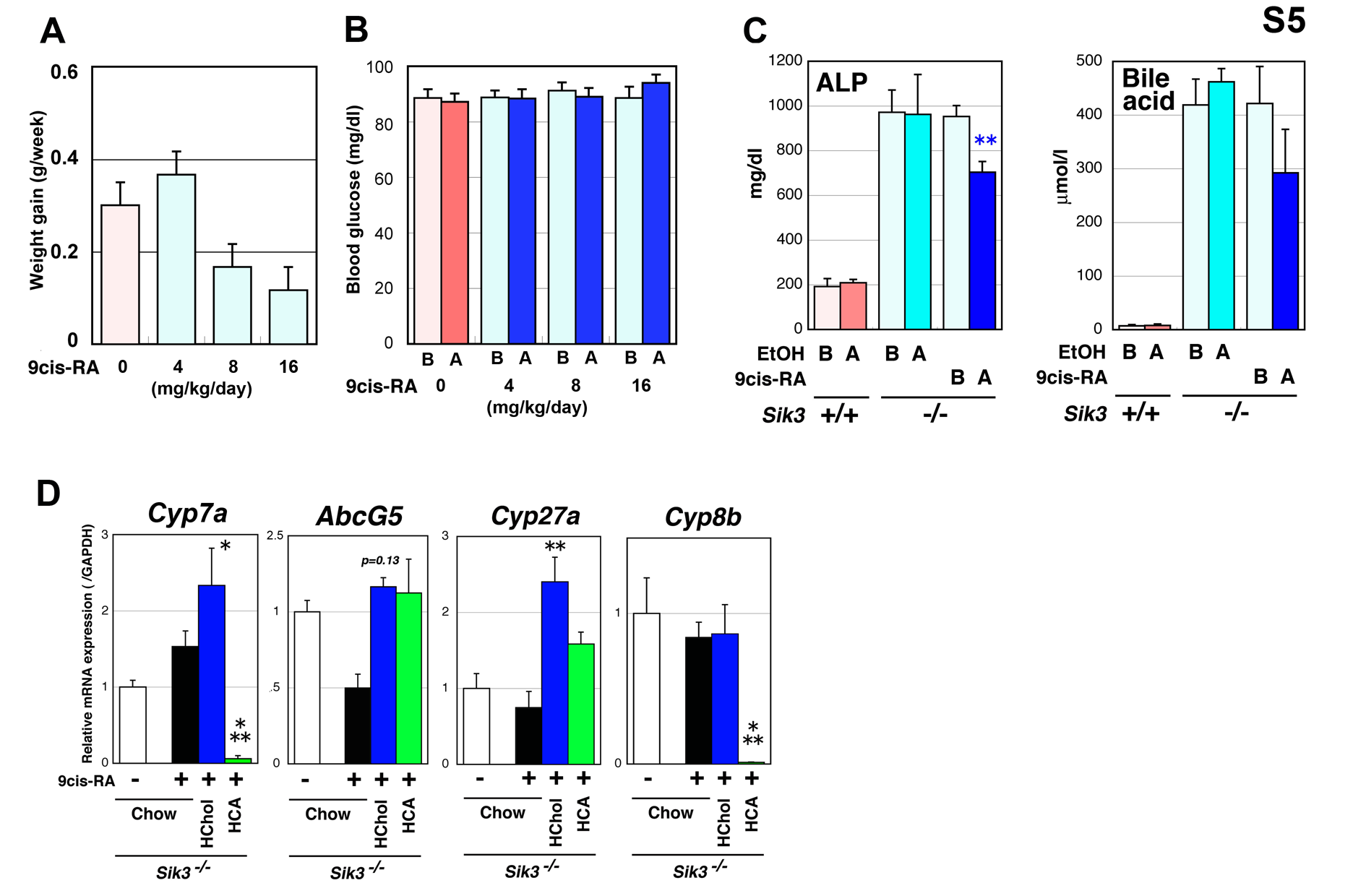

Supplement: Figure S5 — (A) Effect of 9-cis-RA treatment (0–16 mg kg−1·d−1) on the weight gain of wild-type mice (n = 6). (B) Blood glucose levels before and after treatment are indicated by labels as B and A, respectively. (C) The levels of serum ALP and bile acids were measured before (labeled as B) and after (labeled as A) 9-cis-RA treatment (for 9 days: after the analysis shown in Figure 8E). Ethanol (EtOH, 1%) was used as a solvent. Significant differences before and after treatment in the same group (n = 4) are indicated. Although there were no significant fluctuations in the levels of bile acids, their levels decreased in all Sik3 −/− mice after treatment. (D) Effect of 9-cis-RA on gene expression in Sik3−/− mice. At day 7, Sik3 −/− mice treated with 9-cis-RA were grouped into sets of 3 (n = 4) and fed a chow, high-Chol, or high-CA diet for an additional 2 days under continuous RA treatment; mRNA levels in the liver were then examined. Significant differences between the chow and special diet groups are indicated. (TIF) [file pone.0037803.s005.tif]
